# Caught in a TrAP

**DOI:** 10.7554/eLife.11509

**Published:** 2015-10-16

**Authors:** Delfina A Ré, Pablo A Manavella

**Affiliations:** Instituto de Agrobiotecnología del Litoral (IAL), Universidad Nacional del Litoral-CONICET, Santa Fe, Argentina; Instituto de Agrobiotecnología del Litoral (IAL), Universidad Nacional del Litoral-CONICET, Santa Fe, Argentinapablomanavella@ial.santafe-conicet.gov.ar

**Keywords:** epigenetic silencing, viral suppression, geminivirus, histone methyltransferase, TrAP/AL2/AC2, host–virus interaction, Arabidopsis, viruses

## Abstract

Some DNA viruses overcome plant defenses by producing a suppressor protein that blocks the silencing of viral genes.

**Related research article** Castillo-González C, Liu X, Huang C, Zhao C, Ma Z, Hu T, Sun F, Zhou Y, Zhou X, Wang XJ, Zhang X. 2015. Geminivirus-encoded TrAP suppressor inhibits the histone methyltransferase SUVH4/KYP to counter host defense. *eLife* **4**:e06671. doi: 10.7554/eLife.06671

**Image** The virus Transcriptional Activation Protein (TrAP; orange) binds to and inhibits a plant enzyme

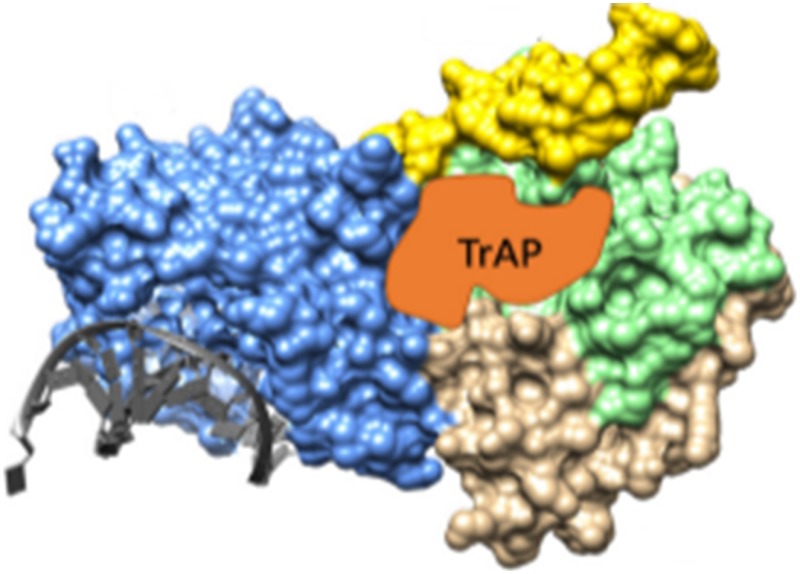

Viruses are parasites that depend on their host to be able to replicate. Animals have mobile immune cells that specialize in detecting and neutralizing viruses. However, plants do not have specialist immune cells so, instead, they rely on mechanisms that are found within all plant cells to block virus replication. Now, in eLife, Xiuren Zhang of Texas A&M University and co-workers – including Claudia Castillo-González as first author – report a new mechanism by which plants can defend themselves against viruses; Zhang and co-workers also report how these viruses manage to counter this defense mechanism (Castillo-González et al., 2015).

When a virus invades a cell and starts to replicate, the production of virus RNA molecules triggers a process known as post-transcriptional gene silencing in which host enzymes convert the RNA molecules into vsiRNAs (virus-derived small interfering RNA molecules). These small RNAs – which can also spread to other cells – are then incorporated into a complex of proteins that represses the expression of the viral genes throughout the plant (Llave, 2010).

The genome of a virus can be made of DNA or RNA and post-transcriptional gene silencing has evolved as a universal defense against both types of viruses. Plants can also defend against DNA viruses using a second process known as transcriptional gene silencing (Pumplin and Voinnet, 2013). This process – which is also used to regulate the expression of a plant’s own genes – can be used to halt virus replication by directly modifying the way DNA is packaged in the cell (Figure 1).

In plants and other eukaryotic organisms, DNA is wrapped around proteins called histones to form a structure called chromatin. Such packing is essential to fit all the genetic material inside the cell nucleus. However, a gene that is in a region of tightly wrapped DNA cannot be expressed. DNA and histones are often modified by the addition of chemical groups known as methyl groups. The pattern of “methylation” in a region of the chromatin influences how tightly it is condensed. Therefore, it rules how highly the genes in that region are expressed (Liu et al., 2010). To activate particular genes, the structure of the chromatin can be relaxed by altering the methylation pattern of its associated histones. However, unlike post-transcriptional gene silencing, researchers do not fully understand how plants use transcriptional gene silencing to defend themselves against viruses.

Geminiviridae is the largest known family of single-stranded DNA viruses in plants. These viruses use host plant histones to pack their DNA and form structures called minichromosomes. Plants control Geminivirus infections by depositing repressive methylation marks into these minichromosomes. It is known that both the Geminivirus DNA and the associated histones are methylated in infected cells (Raja et al., 2008). Remarkably, Castillo-González et al. show that an enzyme called KRYPTONITE binds to the minichromosomes in the plant *Arabidopsis thaliana*. This enzyme – which belongs to the SET domain family of methyltransferases – methylates the virus-associated histones and promotes DNA methylation: the end result is to condense the viral minichromosomes and stop virus replication.

Virtually all plant viruses produce suppressor proteins that block the plant defense mechanisms (Csorba et al., 2015). Geminiviruses produce a suppressor protein called TrAP that inhibits an enzyme that is required to produce the methyl groups needed for methylation. Thus, it was thought that Geminiviruses avoid transcriptional gene silencing by reducing the cell’s pool of methyl groups (Wang et al., 2005). Using cleverly designed in vivo and in vitro experiments Castillo-González et al. found that TrAP interacts with KRYPTONITE and blocks its enzymatic activity to relax the viral chromatin and allow the virus DNA to replicate (Figure 1).

The production of high levels of TrAP in plants also leads to the deregulation of many plant genes whose expression is usually controlled by transcriptional gene silencing. This deregulation could potentially explain the similarities in appearance between TrAP-producing plants and transcriptional gene silencing mutant plants. However, plants that lack a working KRYPTONITE enzyme do not present those physical features, which suggest that TrAP may also block other enzymes belonging to the SET domain family in *A. thaliana*.

In the future, it will be interesting to find out whether some plants are resistant to infection by Geminiviruses because they have methyltransferases that TrAP is unable to bind to. If that turns to be the case, the findings would be of great help to unravel the interactions between plants and viruses and how they have co-evolved. Some Geminiviruses, such as the Maize streak virus infect crops and can cause serious economical losses. The work from Castillo-González et al. might point biotechnologists into new ways to create resistant plants.

**Figure 1.**
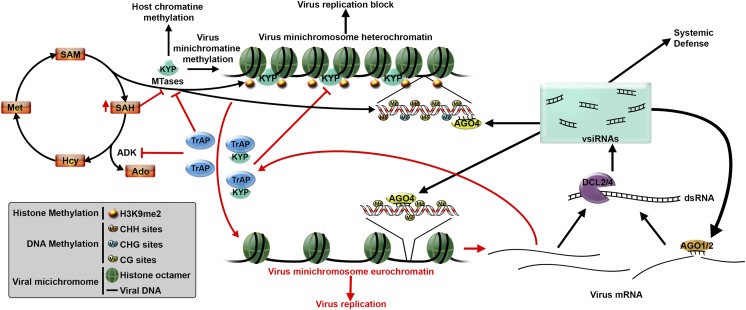
Plant defenses against virus infection can be overcome by a suppressor protein. After infecting a plant cell, a Geminivirus starts to replicate (bottom right). This leads to the production of double stranded RNA molecules, which are processed by a DCL enzyme to produce virus-derived small interfering RNAs (vsiRNAs). These, in turn, trigger two defense mechanisms (black arrows) that aim to block virus replication. The vsiRNAs could be loaded into AGO1 and AGO2 enzymes to silence target viral mRNAs (known as post-transcriptional gene silencing), or could be loaded into AGO4 enzymes to direct DNA methylation (process called transcriptional gene silencing). KRYPTONITE (KYP), or another methyltranserase (MTase), methylates the histones in the viral minichromosome, which also promotes methylation of virus DNA. This results in the minichromosome becoming condensed, which blocks virus replication. However, many viruses produce suppressor proteins, such as TrAP, to counteract these defenses. TrAP blocks transcriptional gene silencing in two ways (red arrows): it inhibits the activity of the ADK enzyme leading to the accumulation of SAH (a molecule that blocks MTase activity) and a reduction in SAM (which is needed for methylation); TrAP can also directly bind to and inhibit the activity of KYP (and perhaps other MTases). Together these two process lead to the de-methylation of the minichromosome, which allows the virus to replicate. Abbreviations: DCL: Dicer-like ribonuclease; AGO: Argonaute; ADK: adenosine kinase; SAH: S-adenosylhomocysteine; SAM: S-adenosyl-methionine.
